# The Patient's Posture in Bed

**Published:** 1919-08-23

**Authors:** 


### DECUBITUS.

### The Patient's Posture in Bed.

By D. M. M.

At a recent examination for a nursing certificate one of the questions put was: "What conclusions might be drawn from a patient's posture in bed?"

The answers to this simple and practical question were so vague and imperfect that a short note on the subject may not be out of place in this paper.

By decubitus is meant the position of a patient in bed. In moderate illness, as in health, it is, as a rule, easy and unconstrained, the patient may arrange the bed-clothes and alter his position when uncomfortable, and will, as a rule, lie on the back or may turn on the side. In the case of the weak, helpless, or unconscious the relaxed body by force of gravity will sink and lie towards the foot of the bed. It should be borne in mind that, even while serious symptoms prevail, being up and about, or being in bed, may sometimes be a matter of temperament. In what is termed the ambulatory or walking type of typhoid fever, for example, a patient may be about with a high temperature and only complain of feeling a little unwell. The position assumed by a patient in bed will be largely decided by comfort. This does not include the attitude in which the position of the patient is effected by the doctor or nurse, and which will be discussed later on.

### Pain and Position.

In the first place, the patient may be unusually quiet, due, it may be, to faintness or intense nausea, since any slight movement of the head may immediately induce sickness. The quietness may be due to slight cerebral hæmorrhage, or to embolism—that is, blocking of some of the brain arteries—or to advanced kidney disease, when, owing to the incomplete removal of waste products from the blood by these organs, drowsiness or even coma may supervene. Sometimes the patient, instead of being very quiet, may exhibit extreme restlessness. A very common cause of this is pain in the abdomen, which may be so tender that the patient lies flat in bed, with the legs drawn up in order that the muscles of the abdomen may become relaxed and so relieve the pressure. A simple test will often indicate the cause of such pain. If the pressure of the warm hand on the abdomen does not increase the discomfort, but rather diminishes it, the pain is not due to peritonitis, but to some local irritation in the abdominal wall or in the bowel as in colic. In this latter the normal contractions of the bowel, which are involuntary and painless in normal conditions, become irregular and unequal, so that spasmodic colic or cramp is induced much on the same principle as cramp occurs in the extremities. In acute peritonitis, on the other hand, almost without exception the abdominal muscles are drawn backwards upon the contents of the abdomen. The reason is that the same nerves supply the peritoneum and the abdominal muscles, and the irritation caused by inflammation of the former also causes the contraction of the muscles of the abdomen. Hence the slightest pressure is resented. This feature is often well marked in appendicitis. Along with the rigid position of the lower part of the body there may be activity of the head and a rolling of it in the pillow.

In acute pleurisy the patient often lies on the side; the particular side may vary and the reasons for its choice prove rather puzzling. The explanation for its selection is, that the position varies with different stages of the disease.

### The Example of Pleurisy.

In the early stages the patient lies with the affected side uppermost; in other words, on the sound side, because it is too painful to lie on the affected side or allow it to touch the bed. When much effusion has occurred the patient lies on the affected side, partly to give the good side free play in breathing, and partly to keep the pressure of liquid off the sound lung. If the patient lies constantly on the back it is frequently a sign of grave and advanced disease, and in such a case a voluntary change to either side often spells some improvement. Patients suffering from heart disease will (as in health) lie more comfortably on one side than on the other—sometimes the right, sometimes the left, for no obvious reason. Another type of patient who prefers to lie on one side is the one with a cavity in the lung. When the cavity lies with its opening below, the secretion flows out, enters the bronchial tube, and by its irritation provokes a constant and distressing cough. If the patient turns over, so that the cavity fills up before the secretion escapes, there is a period of rest, then a large quantity of secretion is expectorated followed by some rest. Once, again, in colic of the bile-ducts, or of the intestine, the patient will lie on the side with the thigh and legs flexed on the abdomen, while the back is arched forward. The same position is often assumed in cerebro-spinal meningitis, except that the back is arched in the opposite direction, to which the term opisthotonus (meaning stretched back) is applied.

### Positions of Distress.

In the paroxysm of asthma, in extreme cases of heart disease, in the effusion of pleurisy and of ascites, and in general dropsy sitting in bed may become uncomfortable, and the patient insists on sitting in a chair, often placing the hands on its arms in order to bring into play some of the additional muscles of respiration. The effusion presses up the diaphragm, embarrasses the lungs and heart, and so makes lying in bed a matter of difficulty. To this the term orthopnœa is applied, from Greek words meaning straight or upright respiration.

Lying on the stomach may be the posture adopted in the case of abdominal pain due to colic or acute gastralgia.

The half-sitting-up position is the one in which the patient is placed in the treatment of general or of pelvic peritonitis. The knees are flexed. Fluids in the abdomen gravitate into the pouch of Douglas—the cavity between the uterus and the rectum—and escape by a drainage-tube or a wick of gauze left there for the purpose. The position is usually termed Fowler's position, from its recommendation by an American surgeon. The prone position may be the one utilised after operations on the tongue and jaws. The object is to permit oozing, should it occur, to escape by the mouth, and so prevent swallowing or choking if the patient is unconscious.

